# Exogenous Cushing Syndrome Caused by a “Herbal” Supplement

**DOI:** 10.1016/j.aace.2022.08.001

**Published:** 2022-08-05

**Authors:** Reema Patel, Sahar Sherf, Ngan Betty Lai, Run Yu

**Affiliations:** 1 Division of Endocrinology, Diabetes & Metabolism, University of California Los Angeles, Los Angeles, California; 2 Division of Endocrinology, Martin Luther King Community Medical Group, Los Angeles, California; 3 Division of Endocrinology, Kaiser Permanente, Santa Rosa, California

**Keywords:** exogenous Cushing syndrome, glucocorticoids, herbal supplements, Artri King, ACTH, adrenocorticotropic hormone, AK, Artri King, FDA, Food and Drug Administration, HPA, hypothalamic-pituitary-adrenal

## Abstract

**Background/Objective:**

Exogenous Cushing syndrome is usually diagnosed in the setting of known glucocorticoid exposure; however, occult glucocorticoid use is possible. We present 2 cases of patients who developed Cushing syndrome while taking Artri King (AK), an over-the-counter “herbal” supplement for joint pains reported to contain glucocorticoids.

**Case Report:**

Patient 1, a 49-year-old woman, reported rapid weight gain, large stretch marks, poor wound healing, and recent diagnoses of type 2 diabetes mellitus and hypertension over a course of 1 year. Her serum am cortisol level was <0.5 μg/dL (reference range, 4.0-22.0 μg/dL) and adrenocorticotropic hormone (ACTH) level was <5 pg/mL (reference range, 5-60 pg/mL). Synthetic glucocorticoid screening revealed a dexamethasone level of 210 ng/dL (reference value < 100 ng/dL) while she was taking AK; 5 days after stopping the supplement, the level was 24 ng/dL (reference value < 20 ng/dL). Patient 2, a 61-year-old woman, presented with weight gain, fatigue, swelling, and recent diagnoses of prediabetes and hypertension over a span of 6 months. Her serum am cortisol level was <1.0 μg/dL (reference range, 8.0-25.0 μg/dL) and ACTH level was <5 pg/mL (reference value < 46 pg/mL). She stopped AK, and 1 month later, her am cortisol level rose to 9.1 μg/dL (reference range, 8.0-25.0 μg/dL) and ACTH level rose to 68 pg/mL (reference value < 46 pg/mL).

**Discussion:**

Supplements containing hidden glucocorticoids and causing Cushing syndrome have been reported in rare cases and can pose a diagnostic challenge for providers.

**Conclusion:**

Exogenous glucocorticoid use because of unregulated herbal supplements should be considered when Cushing syndrome is suspected.

**Highlights**

• Exogenous glucocorticoid use should be considered when Cushing syndrome is suspected
• Exogenous Cushing syndrome is usually diagnosed in the setting of known glucocorticoid exposure; however, occult glucocorticoid use is possible and can complicate its diagnosis
• Markedly low levels of serum cortisol, adrenocorticotropic hormone, and 24-hour urine cortisol in a patient who has features of Cushing syndrome and not of adrenal insufficiency should lead to suspicion of exogenous glucocorticoid exposure
• Medical providers should counsel their patients on the potential dangers of using unregulated over-the-counter supplements

**Clinical Relevance**

We report 2 cases of patients who developed Cushing syndrome while on Artri King (AK), an over-the-counter “herbal” supplement that likely contains glucocorticoids as a hidden ingredient. Given the wide availability of this product, we believe that it is important to share the potentially devastating health effects of using AK.

## Introduction

Cushing syndrome can present as weight gain, particularly around the abdomen; round facies; large striae; proximal muscle weakness; easy bruising; hypertension; and hyperglycemia. It can be caused by adrenocorticotropic hormone (ACTH)-secreting pituitary tumors, ectopic ACTH-secreting tumors, and adrenal tumors or hyperplasia; however, the most common cause of Cushing syndrome is exogenous glucocorticoid use.^1^ In most patients, the diagnosis of exogenous Cushing syndrome is straightforward because they are treated with known systemic glucocorticoids for inflammatory conditions. However, cases of Cushing syndrome with unknown glucocorticoid use because of supplements have been reported. Artri King (AK) (Natural de Mexico LLC), is an over-the-counter supplement marketed as a natural remedy for joint pains. Although AK does not list any glucocorticoids as an ingredient on its label, laboratory analyses by regulatory agencies have reported that their products contain glucocorticoids and advise against their use.^2^^,^^3^ Here, we present 2 cases of patients who we suspect developed exogenous Cushing syndrome because of unknowingly taking glucocorticoids present in this “herbal” supplement.

## Case Report

Patient 1 was a 49-year-old woman who was admitted to the hospital for a left lower extremity wound and cellulitis. She reported gaining 150 pounds of weight, developing fluid retention, new large stretch marks, proximal muscle weakness, poor wound healing, easy bruising, facial puffiness, some minor acne on her chin, and fatigue over the last year. She denied facial flushing or menstrual irregularities; she had previously undergone a hysterectomy. Her medical history also included fibromyalgia, obesity, and recent diagnoses of type 2 diabetes mellitus, hypertension, and central adrenal insufficiency. Adrenal insufficiency had been previously diagnosed based on laboratory results obtained for the workup of possible Cushing syndrome and was thought to have been due to previously being on a course of high-dose steroids. Her medications included hydrocortisone at 10 mg daily, acetaminophen, bumetanide, hydrochlorothiazide, metformin, and pantoprazole. She noted that she occasionally missed her hydrocortisone doses and felt no different from when she took it. She stated that the only other steroid she had ever taken was a 1-month taper of deflazacort (a total dose of 637.5 mg), obtained from a provider in Mexico 1 year ago for fibromyalgia. She denied using any other steroid, including topical, inhaled, or epidural or intra-articular steroid preparations. The only over-the-counter supplement she took was AK, which she obtained from Mexico for joint pain. She had been taking 3 pills on most days for approximately 5 months prior to presentation and stated that this was the only product that effectively managed her joint pain since her month-long course of steroids over a year ago. Physical examination revealed that her blood pressure was 137/58 mm Hg, heart rate was 109 beats/min, and body mass index was 59 kg/m^2^. She had round facies with a prominent dorsocervical fat pad and, as illustrated in the Figure, numerous large, reddish striae over her abdomen, arms, and breasts as well as multiple small ecchymoses on her extremities. She also had an infected left lower extremity wound.

**Fig:**
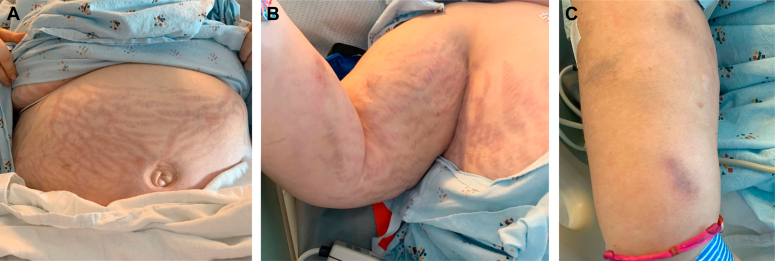
Physical examination findings in patient 1: large pink or red striae over the abdomen, upper extremity, and breast (*A* and *B*) and ecchymosis on the upper extremity (*C*).

Her prior outpatient laboratory workup result was notable for the dexamethasone suppression test, with a cortisol level of 0.6 μg/dL (reference value < 1.8 μg/dL), 24-hour urine free cortisol level of <5 μg/d (reference value < 45 μg/d), serum ACTH level of <5 pg/mL (reference range, 5-60 pg/mL), cortisol level of <0.5 μg/dL (reference range, 4.0-22.0 μg/dL), and synthetic glucocorticoid screening, which was positive for dexamethasone, with a level of 210 ng/dL (reference value < 100 ng/dL), as displayed in the Table.

**Table:** Laboratory Values

Laboratory tests	Patient 1 (on Artri King)	Patient 2 (on Artri King)	Patient 2 (1 mo off Artri King)
am ACTH	<5 pg/mL (reference range, 5-60 pg/mL)	<5 pg/mL (reference range < 46 pg/mL)	68 pg/mL (reference value < 46 pg/mL)
am cortisol	<0.5 μg/dL (reference range, 4.0-22.0 μg/dL)	<1.0 μg/dL (reference range, 8.0-25.0 μg/dL)	9.1 μg/dL (reference range, 8.0-25.0 μg/dL)
24-h urine free cortisol	<5 μg/d (reference value < 45 μg/d)	<5.6 μg/d (reference value < 50 μg/d)	…
Dexamethasone suppression test	0.6 μg/dL (reference value < 1.8 μg/dL)	…	…
Synthetic glucocorticoid screening	Dexamethasone 210 ng/dL (reference value <100 ng/dL)	…	…
Dexamethasone	24 ng/dL (reference value < 20 ng/dL)	…	…

Abbreviations: ACTH = adrenocorticotropic hormone.

The patient was instructed to stop the AK supplement and continue hydrocortisone. Five days after discontinuing AK, her dexamethasone level was 24 ng/dL (reference value < 20 ng/dL). She was monitored as an outpatient for improvement of her hypothalamic-pituitary-adrenal (HPA) axis and tapering of hydrocortisone. Over a course of 2 weeks, she lost 14 pounds of weight.

Patient 2 was a 61-year-old woman who presented to the endocrinology clinic for the evaluation of a weight gain of 16 pounds, fatigue, swelling, and mild generalized weakness over the last 6 months. She denied any stretch marks or easy bruising. She developed new-onset prediabetes and hypertension during this period. Her medical history also included osteoarthritis, asthma, and hypothyroidism. Her medications included lisinopril, simvastatin, levothyroxine, and duloxetine. She denied using any oral or injectable steroids but had used 2 tubes (3.5 g each) of a neomycin-polymyxin-b-dexamethasone (3.5 mg/mL-10 000 units/g-0.1%) ophthalmic ointment over the last 9 months. She had stopped using the ophthalmic ointment 3 weeks prior to presentation. She was also taking over-the-counter AK supplements—2 tablets daily from Monday through Friday—which she had purchased from Mexico, for the last 2 years.

Physical examination revealed a blood pressure of 123/58 mm Hg, a body mass index of 26.6 kg/m^2^, and pitting lower extremity edema. She did not have any notable round facies, dorsocervical fat pad, striae, ecchymoses, or proximal muscle weakness.

As shown in the Table, her laboratory workup showed an am serum cortisol level of <1.0 μg/dL (reference range, 8.0-25.0 μg/dL), ACTH level of <5 pg/mL (reference value < 46 pg/mL), and 24-hour urine free cortisol level of <5.6 μg/d (reference value < 50 μg/d). Repeat am cortisol and ACTH levels remained undetectable 5 weeks later.

She was instructed to stop taking AK, and after 1 month, she reported a weight loss of 10 pounds and improved blood pressure. Additionally, her am cortisol level rose to 9.1 μg/dL (reference range, 8.0-25.0 μg/dL) and ACTH level rose to 68 pg/mL (reference value < 46 pg/mL). She was started on hydrocortisone at 5 mg daily because of nausea that started after stopping AK.

## Discussion

Both our patients presented with weight gain and recently diagnosed hyperglycemia and hypertension, raising concern for hypercortisolism. Laboratory testing revealed suppression of their HPA axes; therefore, we suspected possible exogenous glucocorticoid use.

Oral, injected, topical, and inhaled glucocorticoids have all been implicated in cases of exogenous Cushing syndrome.^4^, ^5^, ^6^, ^7^ Because of its intrinsic glucocorticoid activity, megestrol acetate has also been shown to cause Cushing syndrome at high doses.^8^ Itraconazole and ritonavir, which inhibit the cytochrome P450 3A4 metabolism of glucocorticoids, can delay the clearance of some glucocorticoids and cause Cushing syndrome even with exposure to lower doses of glucocorticoids.^9^^,^^10^ Surreptitious and occult glucocorticoid use has also been reported.^11^, ^12^, ^13^

No clear offending agents were noted to be taken by our patients at the time of presentation; however, both were taking AK. The product label lists glucosamina, condroitina, colageno, vitamina C, curcuma, ortiga, omega 3, and MSM (which we believe translates to glucosamine, chondroitin, collagen, vitamin C, curcumin, nettle, omega-3 fatty acids, and methylsulfonylmethane in English) as its ingredients. Still, we suspected it to be the source of glucocorticoids based on a process of elimination. In addition, an online search of AK revealed news articles stating that testing of this company’s products found hidden ingredients, including dexamethasone, methylprednisolone, and diclofenac.^2^ We believe that patient 1 developed Cushing syndrome after her initial course of deflazacort and continued to have signs and symptoms of hypercortisolism later, with a suppressed HPA axis, because of taking AK. Patient 2 had recently used an ophthalmic steroid ointment; however, given the lack of improvement after discontinuing it, we thought that the ointment was unlikely to have contributed. Notably, she appeared to have a less striking presentation than patient 1, likely because of lower overall glucocorticoid exposure while on AK alone. Discontinuing AK led to weight loss in both the patients; however, because of prolonged suppression of their HPA axes, they currently require replacement glucocorticoid doses. Although we were not able to test the actual supplements used by our patients to confirm their contents, the decrease in dexamethasone level in patient 1 after discontinuing AK suggests that the likely source of dexamethasone was AK.

The U.S. Food and Drug Administration (FDA) has noted a growing trend of dietary supplements with hidden drugs or ingredients.^3^ Supplements are classified as food products, not medicines; therefore, they are not subject to the strict regulatory standards of the FDA. Many over-the-counter “adrenal support” supplements have been found to contain steroids, and even supplements containing adrenal extracts from animals are sold to the general public with claims of aiding adrenal function.^14^ AK is part of a line of multiple products for joint pains and arthritis. These supplements are promoted as being “all natural” and are available for sale through various retailers. Since the management of the cases of the patients presented here, the FDA has issued a public notification advising consumers not to purchase AK because their laboratory analysis revealed dexamethasone and diclofenac as unlisted ingredients.^3^ No further details were given about the specifics of how the FDA conducted the testing of the AK products. To our knowledge, there are no prior reported cases of an AK supplement causing Cushing syndrome published in the English medical literature. We found 1 medical publication written in Spanish that details a case of Cushing syndrome in a patient taking an AK supplement.^15^ Given the availability of AK to patients in the United States, we believe that it is important to describe the potentially devastating health effects of using this product in the English literature as well.

## Conclusion

For cases of suspected Cushing syndrome in which glucocorticoid use is not revealed based on the patient’s history, laboratory evaluation can offer some clues. Markedly low levels of serum cortisol, ACTH, and 24-hour urine cortisol in a patient who does not appear to have adrenal insufficiency should lead to suspicion of exogenous glucocorticoid exposure. Furthermore, synthetic glucocorticoid screening can help confirm exogenous glucocorticoid use. Our 2 cases emphasize the possibility of developing Cushing syndrome by unknowingly ingesting glucocorticoids from a misleadingly labeled over-the-counter “herbal” supplement. Patients should be warned about the use of AK products, and medical providers should counsel their patients on the potential dangers of using unregulated over-the-counter supplements.

## Disclosure

The authors have no multiplicity of interest to disclose.
